# Clinical Evaluation of a Low Cost, In-House Developed Real-Time RT-PCR Human Immunodeficiency Virus Type 1 (HIV-1) Quantitation Assay for HIV-1 Infected Patients

**DOI:** 10.1371/journal.pone.0089826

**Published:** 2014-03-06

**Authors:** Palvinder Kaur, Wei Xin Khong, Sue Yuen Wee, Eng Lee Tan, Juergen Pipper, Evelyn Koay, Kah Ying Ng, Joe Kwan Yap, Kuan Kiat Chew, Mei Ting Tan, Yee Sin Leo, Masafumi Inoue, Oon Tek Ng

**Affiliations:** 1 Institute of Infectious Disease and Epidemiology, Communicable Disease Centre, Tan Tock Seng Hospital, Singapore, Singapore; 2 Experimental Therapeutics Centre, Agency for Science, Technology and Research (A*STAR), 31 Biopolis Way, Nanos #03-01, Singapore, Singapore; 3 Centre for Biomedical and Life Sciences, Singapore Polytechnic, Singapore, Singapore; 4 Department of Paediatrics, University Children's Medical Institute, National University Hospital, Singapore; 5 Institute of Bioengineering and Nanotechnology, 31 Biopolis Way, The Nanos, Singapore, Singapore; 6 Molecular Diagnosis Centre, Department of Laboratory Medicine, National University Hospital, Singapore; 7 Department of Pathology, Yong Loo Lin School of Medicine, National University of Singapore, Singapore; 8 Yong Loo Lin School of Medicine, National University of Singapore, Singapore; 9 Lee Kong Chian School Of Medicine, Nanyang Technological University, Singapore; University of Missouri-Kansas City, United States of America

## Abstract

**Objectives:**

HIV-1 viral quantitation is essential for treatment monitoring. An in-house assay would decrease financial barriers to access.

**Materials and Methods:**

A real-time competitive RT-PCR in house assay (Sing-IH) was developed in Singapore. Using HXB2 as reference, the assay's primers and probes were designed to generate a 183-bp product that overlaps a portion of the LTR region and *gag* region. A competitive internal control (IC) was included in each assay to monitor false negative results due to inhibition or human error. Clinical evaluation was performed on 249 HIV-1 positive patient samples in comparison with the commercially available Generic HIV Viral Load assay. Correlation and agreement of results were assessed for plasma HIV-1 quantification with both assays.

**Results:**

The assay has a lower limit of detection equivalent to 126 copies/mL of HIV-1 RNA and a linear range of detection from 100–1000000 copies/mL. Comparative analysis with reference to the Generic assay demonstrated good agreement between both assays with a mean difference of 0.22 log_10_ copies/mL and 98.8% of values within 1 log_10_ copies/mL range. Furthermore, the Sing-IH assay can quantify HIV-1 group M subtypes A–H and group N isolates adequately, making it highly suitable for our region, where subtype B and CRF01_AE predominate.

**Conclusions:**

With a significantly lower running cost compared to commercially available assays, the broadly sensitive Sing-IH assay could help to overcome the cost barriers and serve as a useful addition to the currently limited HIV viral load assay options for resource-limited settings.

## Introduction

With an estimated 34 million people living with HIV worldwide and 1.7 million deaths in 2011 [1], HIV-1 is a global pandemic that remains one of the world's most serious health challenges. Currently, more than 10 million individuals receiving HIV-1 antiretroviral therapy, many of which living in low- and middle-income countries [2]. HIV-1 viral quantitation is essential for treatment monitoring [3]–[6].

HIV-1 viral quantitation remains limited in many affected regions, especially in resource-limited settings [7]. Where available, the running cost of commercially available viral load assays is prohibitively expensive: ranging from US$20–$160 per viral load test. The prohibitive high running cost of commercially available viral load assays has impeded their routine application in most resource-limited settings [7], [9].

In the absence of HIV-1 viral quantitation, treatment decisions based on CD4 cell counts and clinical staging can result in delays of treatment, misdiagnoses and evolution of drug-resistant virus [8]–[10]. By far, no surrogate marker has been able to replace the viral load for monitoring treatment response, thus rendering HIV-1 viral quantitation an essential tool for treatment monitoring [6], [11]. In light of the critical role of viral load monitoring, World Health Organization (WHO) strongly recommends implementation of routine viral load monitoring in resource-limited settings, with viral load tests at both six and twelve months after treatment initiation, and subsequently at least every twelve months thereafter [12].

We developed a viral load assay with internal control, targeting a 189-bp region starting from 3′ end of the LTR region to part of 5′ of the *gag* region of HIV-1 virus (PCT Patent Application No. PCT/SG2010/000257). We detailed the results of evaluation of the Sing-IH, an in-house developed probe-based real-time PCR (RT-PCR) for the rapid detection of HIV-1 RNA in plasma samples. Clinical evaluation of the assay was performed comparing the results of the in-house assay with results generated from the Generic HIV Viral Load assay in 249 patient samples [13], [14]. Detection of HIV-1 subtypes was evaluated against a genotype panel obtained from the National Institute of Biological Standards and Controls (NIBSC). Additionally, we present results of performance of the Sing-IH in the Treat-Asia Quality Assurance Scheme (TAQAS) for HIV-1 Viral Load Testing.

## Materials and Methods

### Ethics Statement

The study protocol was reviewed and approved by the ethics institutional review board of the National Healthcare Group in Singapore. The participants provided their written informed consent to participate in this study.

### Samples and Extraction of Viral RNA From Clinical Specimens

Plasma samples were collected from HIV-1 infected patients (both treatment-naïve and treatment-experienced, n = 249) at the Communicable Disease Centre, Singapore. Whole blood samples collected in EDTA tubes were centrifuged at 1500 g for 15 min within 6 hours of collection and plasma obtained was stored at −80°C until use. Automated viral RNA extraction method was performed using the Roche Magna Pure Compact System (Roche Applied Science, Switzerland). Prior to extraction, 1 mL of frozen plasma was thawed at room temperature, and ultra-centrifuged at 25000 g for 1 h at 4°C. 600 µl of supernatant was discarded and the remaining 400 µl of plasma containing the viral pellet was used for RNA extraction using the Magna Pure Compact Nucleic Acid Isolation kit (Roche Applied Science, Switzerland), following the manufacturer's protocol. RNA was then eluted in 50 µL of elution buffer and used for both Sing-IH and Generic HIV Viral Load assay.

### Oligonucleotide Design

In house primers and probes were designed using alignment data referenced from the ‘HIV Sequence Compendium 2008’, HIV Los Alamos National Laboratory Database [15]. The Sing-IH primers target a consensus region at the 3′ end of LTR gene to part of the 5′ end of the gag gene. The forward primer (Gag183UF) is an overlap of the 3′ LTR region and Lys tRNA primer-binding site. The reverse primer (Gag187LR) is an overlap of the region that codes for the packaging loop and the start of 5′ *gag* gene. The Taqman detection probe (Gag187P), which hybridizes to the 183-bp PCR product, is situated at the start of the packaging loop. The detection probe had a fluorescence reporter, 6-carboxyfluorescein (6-FAM) attached at the 5′ end and Black Hole Quencher 1 (BHQ-1) linked to the 3′end. The nucleotide sequences of primers, probes and internal control are shown in Table 1. Primers and probes were derived from the gene sequence of HIV-1 genotype B and AE.

**Table 1:** Primers and probes used to detect the HIV-1 *gag* region and internal control (IC) molecules.

Primer/Probe	Type	Nucleotide sequence (5′–3′)
Gag183UF	Sense primer	CTAGCAGTGGCGCCCGAACAG
Gag187LR	Antisense	CCATCTCTCTCCTTCTAGCCTCCGCTAGTCA
Gag187P	Detection probe	[FAM]-TCTCTCGACGCAGGACTCGGCTTGCTG-[BHQ1]
Gag187ICP2	Probe for IC	[Texas]-AGGTCGGGTGGGCGGGTCGTTA-[BHQ2]

### Internal Control (IC)

A random sequence of a competitive internal control (IC) was designed to incorporate a unique probe-binding site different from the HIV-1 target molecules. This chimerical single strand DNA which hybridizes the primers gag183U and gag187L was co-amplified in each round of assay as described below. Calibration experiments showed no interference with target HIV-1 detection.

100 copies of a DNA-based amplification control were spiked into each reactions of the real-time PCR assay to monitor the performance of the PCR and to detect inhibition. A sample with a Ct value of the IC delayed by more than 2 cycles was considered invalid due to PCR inhibition and repeated.

### Analytical Standards

AcroMetrix HIV-1 RNA Panel (Optiquant quantification panel HIV-1 RNA, Acrometrix Inc., CA, USA), a commercial standard calibrated against the WHO international HIV RNA standard were used as analytical standards. The AcroMetrix standards were consist of HIV-1 standards at concentrations of 10000000, 1000000, 100000, 10000, 1000 IU/mL. The conversion factor applied was 2 IU/copy as described in manufacturer's protocol [16].

A subtype reference panel containing HIV-1 subtypes M (Subtypes A–D, AE, F, G, AG), N and O was obtained from National Institute of Biological Standards and Control (NIBSC). Viral loads were determined using the Sing-IH and Generic HIV Viral Load assays.

### Real-time Reverse Transcription PCR

Real-time PCR was performed using Superscript™ III Platinum One Step Quantitative RT-PCR System Master Mix reagents (Life Technologies, CA, USA) in a total volume of 25 µL containing 10 µL RNA sample, 100 copies of IC molecules, a final concentration of 0.3 µM of HIV-1 forward/reverse primers, and both probes (FAM/ROX) at concentrations of 0.1 µM each. Thermal Cycling was performed in CFX96 Touch™ Real-Time PCR Detection System (Bio-Rad Laboratories, CA, USA) using the following steps: reverse transcription at 55°C for 30 min and initial denaturation at 95°C for 2.5 min, followed by 45 cycles of denaturation at 95°C for 17 s, annealing at 67°C for 31 s and extension at 70°C for 32 s. Fluorescence detection was read at 70°C of each cycle.

The automated baseline mode was used for cycle threshold (Ct) determination for data analysis for both the target and IC. Single determinations were performed to mimic routine clinical use. Each sample run included 2 negative controls to exclude contamination. To control for the extraction and reverse transcription processes, HIV-1 AcroMetrix standards and positive control were processed along with patient specimens in each run. According to the manufacturer's manual, the slope of the HIV-1 Acrometrix standards should read between −3.6 and −2.9; while the positive control should measure within 3.7±0.4 log_10_ copies/mL. In the event where any of the standard or control results exceeded the established validity range of the sample, all of the specimens and controls from the run were retested.

Standard curves generated from duplicates of the AcroMetrixHIV-1 RNA Panel were used to determine the copies of HIV-1 RNA per reaction. Final concentration of viral RNA copies per mL was calculated.

### Commercial Viral Load Assay

In house viral load assays were validated against the commercially available Generic HIV Viral Load assay (Biocentric, Badol, France) according to manufacturer's instructions. Standard curve was established with the Optiquant quantification HIV-1 RNA panel included in the kit.

### Statistical Analysis

Microsoft™ Excel 2000 (Microsoft Corporation, USA), Graphpad Prism Version 5.00 for Mac (Graphpad Software, San Diego, USA), SPSS (SPSS Statistics for Windows, Version 17.0, USA) and StatsDirect (StatsDirect Ltd, UK) were used for statistical analyses described in this study. Bland-Altman plots were used to determine the agreement of the in house HIV assay with the commercial assay [17], [18]. Probit regression analysis was used to determine the theoretical lower limit of quantitation.

### Treat Asia Quality Assurance Scheme (TAQAS)

Sing-IH was enrolled for the Treat Asia Quality Assurance Scheme (TAQAS) for the Human Immunodeficiency Virus (HIV) RNA Viral Load External Quality Assessment Scheme (EQAS) proficiency test [19]. Five HIV-1 plasma samples with unknown viral loads were obtained from TAQAS and subjected to the Sing-IH Assay protocol. Viral loads were then reported back to TAQAS.

## Results

### Linearity of dynamic range

**Figure 1:**
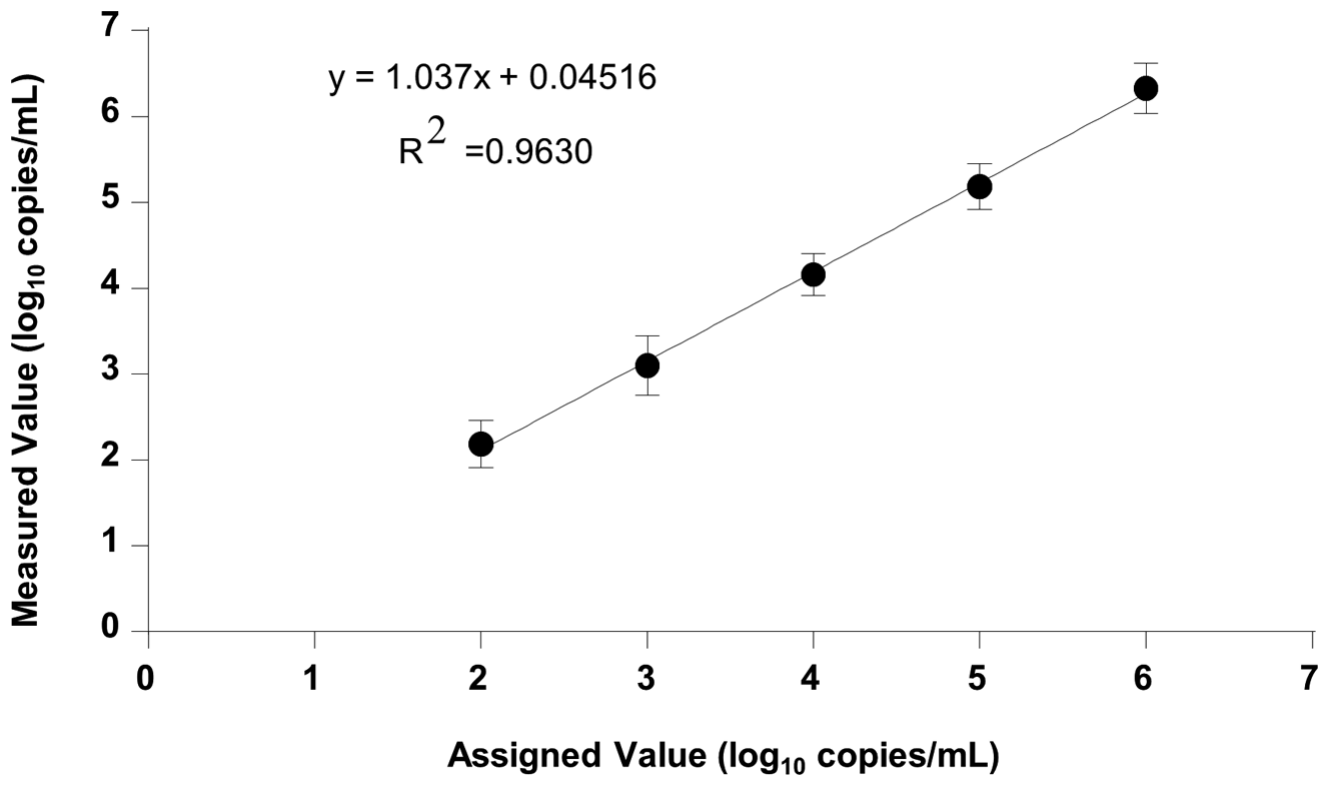
Plot of results from a linearity experiment to determine reportable range.

The linearity of Sing-IH assay was established using a five-member HIV panel from 19 replicate assays. Assigned values were plotted against measured values (Figure 1). The reportable linear range of the assay is 100 copies to 1000000 copies. Linear regression analysis gave an equation of y = 1.037×+0.04516. The R^2^ values of 0.9630 showed good linearity throughout the range tested.

### Analytical sensitivity

**Figure 2:**
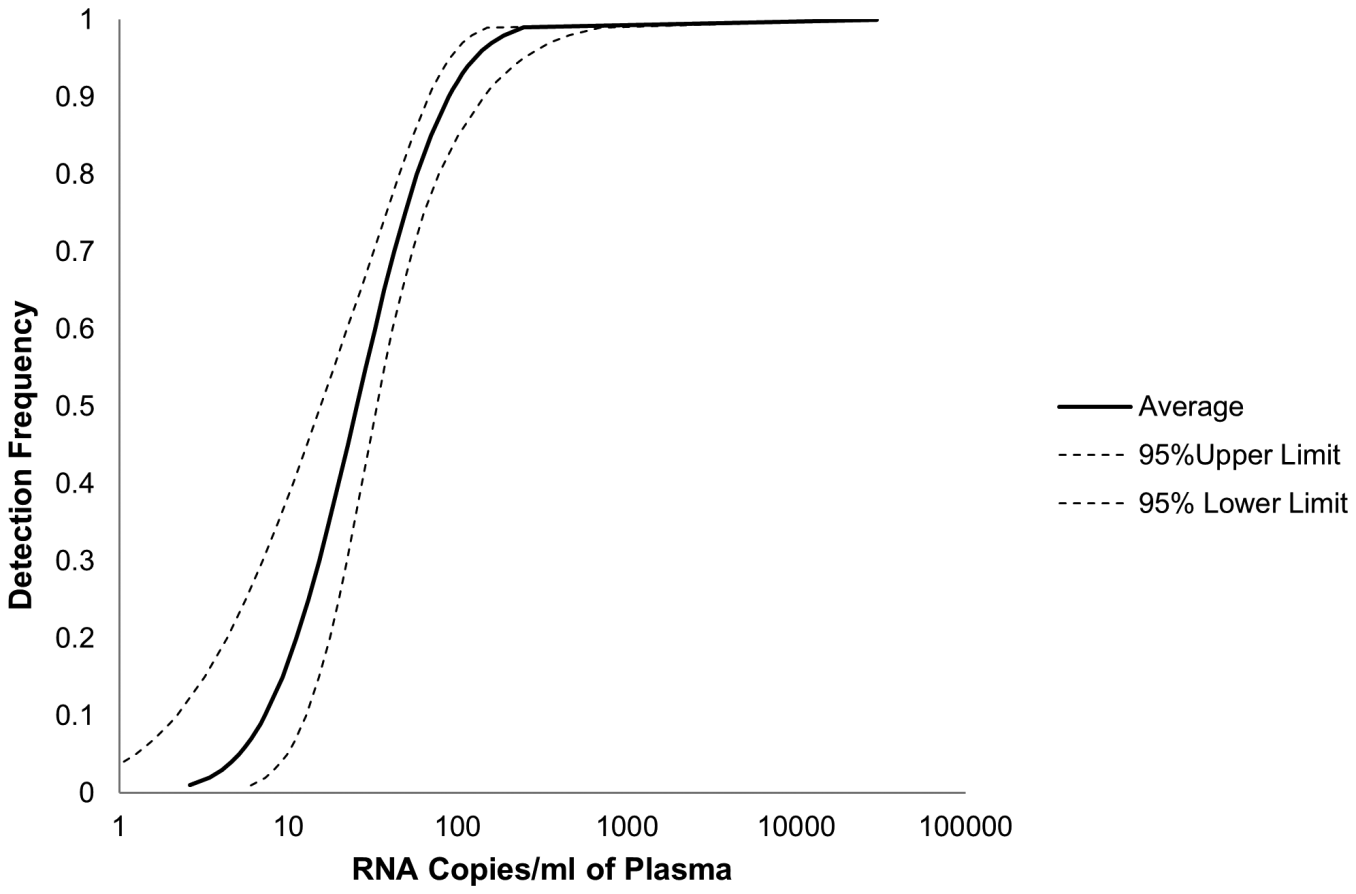
Probit regression analysis to determine the limit of detection of the in-house HIV viral load assay. 125.5 copies/mL (2.10 log_10_ copies/mL) at 95% CI (range 89.6–243.9 copies/mL; 1.95–2.39 log_10_ copies/mL).

A range of 8 serially diluted HIV-1 samples was used for statistically precise determination of the detection limit. Specifically, a well-quantified HIV-1 sample was diluted to a final concentration of 1000000, 100000, 10000, 1000, 150, 100, 50, 25, copies/mL using Acrometrix EDTA plasma dilution matrix prior to RNA preparation and tested in 19 replicates with exception for the lower copy number samples. For RNA samples with lower copy numbers, (150, 100, 50, 25 copies/mL) more replicates (n = 40) were subjected to analysis to ensure the accuracy of statistical analysis. The numbers of positive and negative reactions obtained with each of the 8 RNA concentrations were subjected to probit regression analysis (Figure 2) to calculate the probability of achieving a positive result at any RNA concentration within the range of 0 to 1000000 copies per ml of plasma. At a concentration of 126 copies/mL (corresponding to 2.099 log_10_ copies/mL) detection probability was 95% or greater (1.952–2.387 log copies/mL or 90–244 copies/mL) for the Sing-IH assay (Figure 2). The detection limit of Sing-IH for our routine blood samples was therefore set at 126 copies/mL.

### Precision

Inter-assay accuracy was assessed with 2 replicates of known HIV-1 RNA concentrations at 100, 1000 and 10000 copies/mL in 9 batched experiments. The mean of the measured viral loads were 14374.91, 1275.97 and 154.64 copies/mL, respectively (Table 2). The CVs were 5.19%, 9.70% and 8.23%, with SD of 0.22, 0.30 and 0.18 for the 3 concentrations respectively.

**Table 2:** Inter-assay precision.

Known Concentration (Copies/mL)	Mean Concentration copies/mL	Mean Concentration Log10 Copies/mL	% CV	SD	95% CI (Log10 Viral Load)	Log10 Change 95% CI
10000.00	14374.91	4.16	5.19	0.22	3.976–4.651	0.68
1000.00	1275.97	3.11	9.70	0.30	2.728–3.760	1.03
100.00	154.64	2.19	8.23	0.18	2.009–2.577	1.59

Two replicates at 3 concentrations were performed in batch of 9 experiments each.

Intra-assay accuracy was assessed with 18 replicates of known HIV-1 RNA concentrations of 6300 and 100 copies/mL in a single experiment. Mean measured viral loads were 5834.45 and 173.78 copies/mL respectively (Table 3). CVs were of 5.60% and 5.98% with SDs of 0.21 and 0.13 respectively.

**Table 3:** Intra-assay precision.

Known Concentration (Copies/mL)	Mean Concentration copies/mL	Mean Concentration Log10 Copies/mL	% CV	SD	95% CI (Log10 Viral Load)	Log10 Change 95% CI
6300.00	5834.45	3.77	5.60	0.21	3.387–4.023	0.20
100.00	173.78	2.24	5.98	0.13	2.008–2.383	0.28

Eighteen replicates at 2 concentrations were performed in a single experiment.

### Quantitation of HIV-1 subtypes

**Figure 3:**
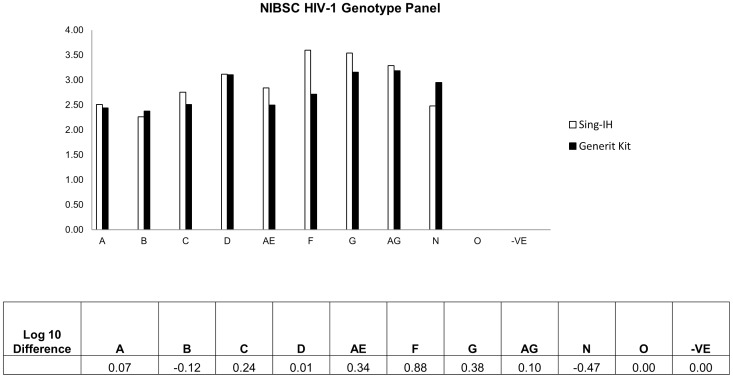
Verification of NIBSC subtype panel with SING-IH assay and Generic HIV-1 Viral Load. Viral loads were determined for plasma containing HIV-1 from subgroup A, B, C, D, E, F, G, AG, N and O using both in-house HIV viral load assay and commercially-available Generic HIV-1 Viral Load. The log_10_ differences in viral loads are plotted at the bottom. Letters at x axis represent the HIV strains present in the tested samples. ND- non-detectable.

The ability of SING-IH to quantitate a diversity of HIV-1 subtypes was assessed by qualitatively assaying the NIBSC 1st International Reference Panel for HIV-1 RNA Genotypes. Our results showed that both Sing-IH and Generic HIV Viral Load assay were able to detect HIV-1 viruses from group M (A, B, C, D, AE, F, G, AG), and group N genotypes (Figure 3). The differences in viral loads between both assays were also calculated (Figure 3). However, as expected, both assays were unable to detect samples from Group O.

### Evaluation of the in-house assay with 249 patient samples

Viral loads for 249 HIV-1 infected plasma samples were obtained using the SING-IH assay and validated against the commercially available Generic HIV Viral Load assay. Among these 249 plasma samples, 1 sample was detected by SING-IH assay (1.03 log_10_ copies/mL) but not Generic HIV Viral Load assay. Due to the fact that the quantitative value was below the limit of detection of the Generic HIV Viral Load assay, this sample was excluded from the Bland-Altman analysis.

**Figure 4:**
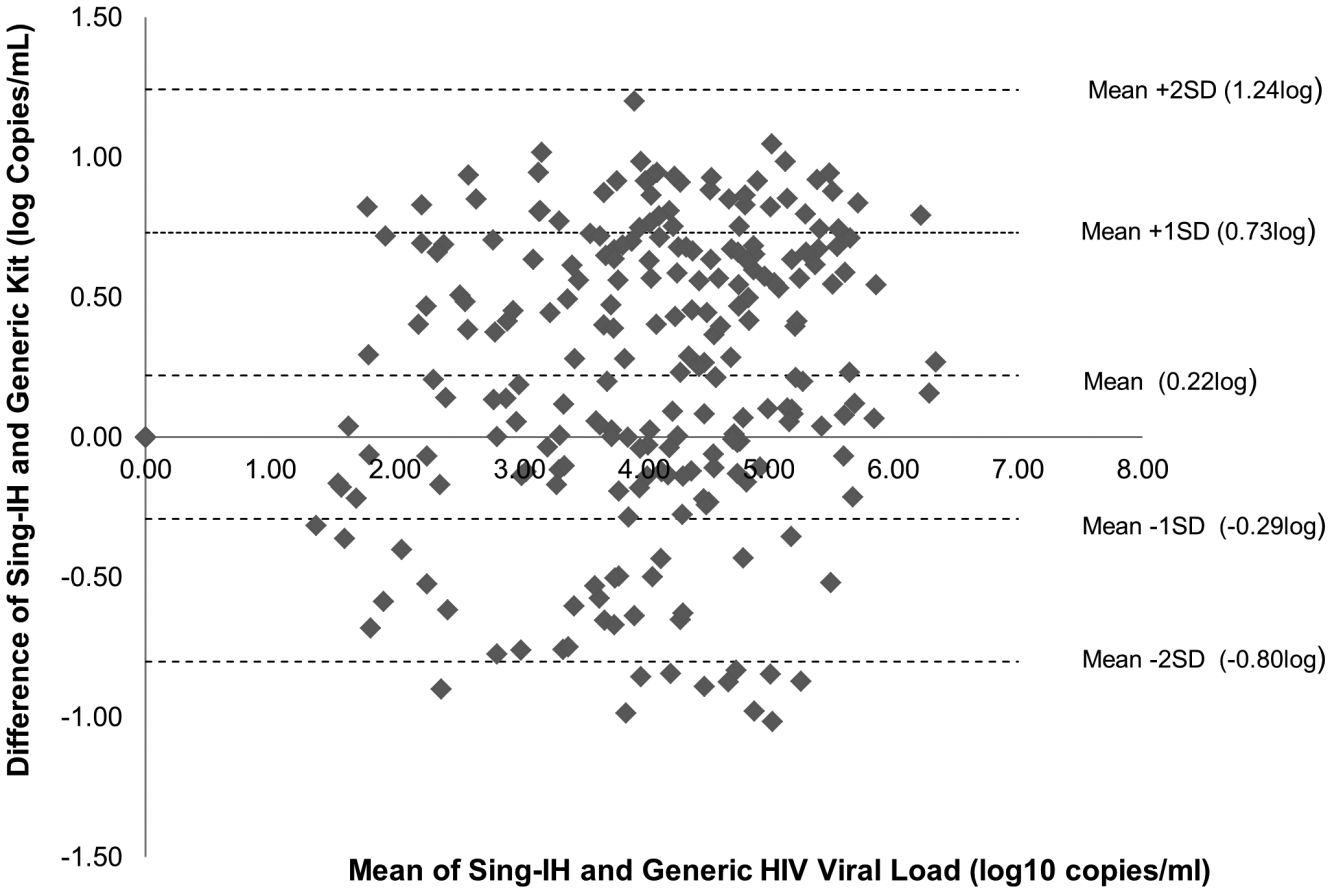
Bland-Altman bias plot for in-house HIV viral load assay and Generic HIV-1 Viral Load. Two-hundred and forty-nine patient specimens were tested using both in-house HIV viral load assay and Generic HIV-1 Viral Load. According to the Bland-Altman analysis, 63.31% of the sample readouts were within 0.5 log_10_, 35.48% were within 0.5 to 1 log_10_ and 1.21% showed greater than 1 log_10_ difference.

**Figure 5:**
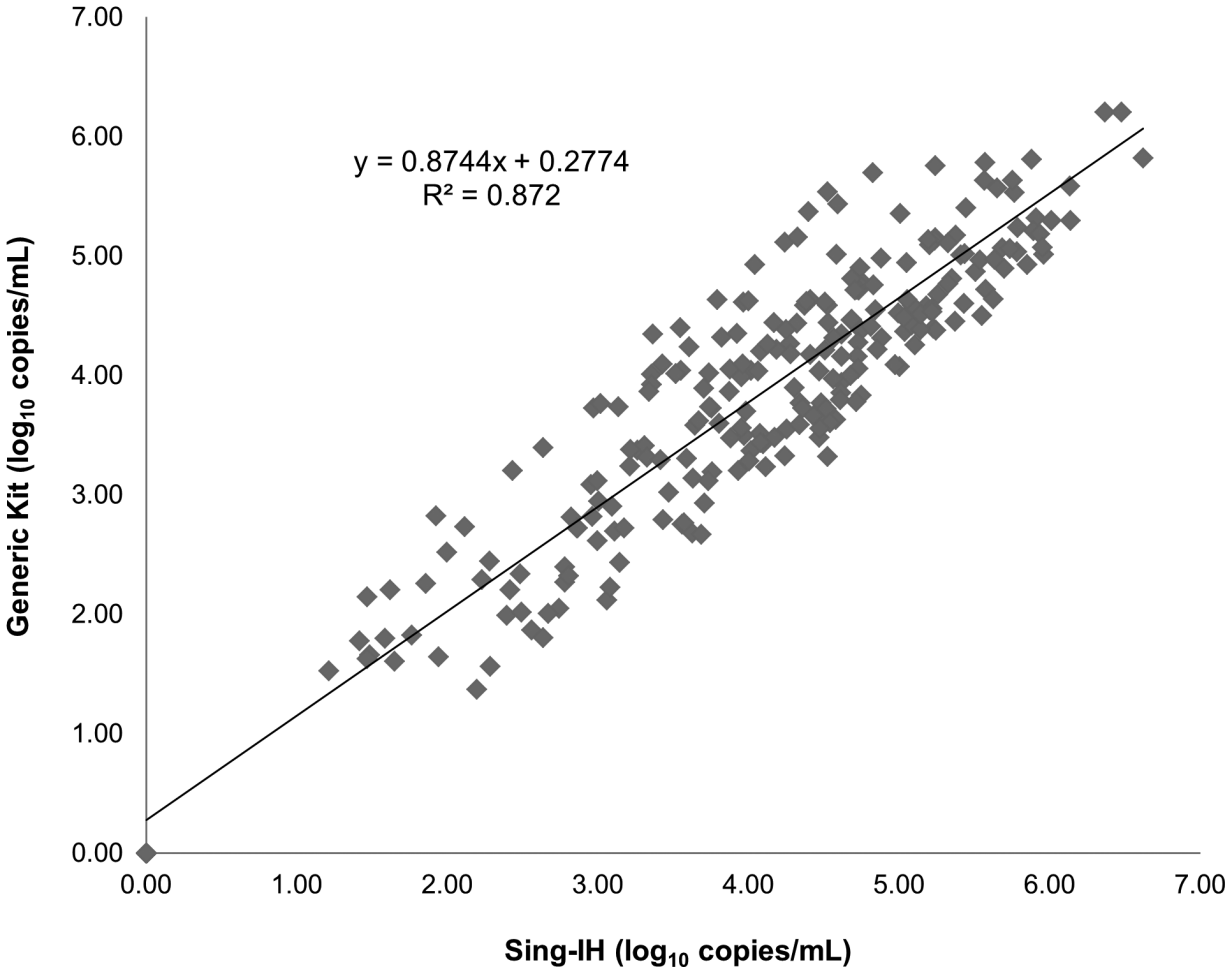
*xy* scatter plot for in-house HIV viral load assay and Generic HIV-1 Viral Load. Linear regression analysis gives the equation y = 0.8744×+0.2774 with a correlation coefficient of 0.87, indicating good correlation. The intra-class correlation coefficient between the 2 assays was 0.87, with no clinically significant bias was observed for Sing-IH and Generic HIV-1 Viral Load assays.

Bland-Altman analysis showed that the mean difference between Sing-IH and Generic assay was 0.22 log_10_ copies/mL (1.20 to −1.01 log_10_ copies/mL, P<0.001), indicating an excellent agreement between Sing-IH and the commercial assay (Figure 4). According to the analysis, 63.31% of the sample readouts fell within 0.5 log_10_, 35.48% were within 0.5 to 1 log and 1.21% showed greater than 1 log difference. The intra-class correlation coefficient between the 2 assays was 0.87 (Figure 5), with no clinically significant bias observed for Sing-IH and Generic HIV-1 Viral Load assays.

Overall, sensitivity and specificity of SING-IH were 99.6% and 100% respectively, using the Generic HIV Viral Load assay as reference.

### Human Immunodeficiency Virus (HIV) RNA Viral Load External Quality Assessment Scheme (EQAS) Proficiency Test [Panel ID: 2013-06-26]

Comparisons of Sing-IH and TAQAS results were made using 5 HIV-1 samples with unknown viral load. A mean difference of 0.16 log_10_ copies/mL with SD of 0.13 log_10_ copies/mL was obtained (Table 4).

**Table 4:** HIV-1 RNA Viral Load External Quality Assessment Scheme (EQAS) Proficiency Test [Panel ID: 2013-06-26].

SAMPLES	Sing-IH Results (log copies/ml)	NRL Results (log10 Copies/ml)	Difference (log_10_ Copies/ml)
**A**	4.35	4.07	0.28
**B**	3.28	3.29	−0.01
**C**	2.43	2.37	0.06
**D**	4.36	4.07	0.29
**E**	5.35	5.18	0.17

## Discussion

HIV-1 viral load monitoring remains grossly underused due to its prohibitive high cost in many settings in Southeast Asia [7], [9]. Recognizing the biggest hurdle to widespread access to viral load test, the Sing-IH assay was developed with the aim to provide an affordable, highly sensitive alternative for routine clinical use in regions where subtype B and CRF01_AE predominate [20]–[22].

To our knowledge, 3 comparisons have been carried out so far to evaluate the performance of Biocentric Generic HIV Viral Load, all of which demonstrated excellent correlation when compared against various commercially available assays [13], [14], [23]. The assay's excellent clinical performance and its wide spread use in resource-limited settings prompted us to chose the Generic assay as the reference method for evaluation of our in-house developed SING-IH assay. In this study, we showed that Sing-IH assay is comparable to Biocentric Generic HIV Viral Load assay in quantitating 249 patient samples. Moreover, the Sing-IH assay demonstrated comparable clinical performance with other in-house assay currently in clinical use, such as the assay developed by Drosten *et al.*, which was validated against 3 commercial assays on 1487 patient samples from Brazil, India, South Africa and Germany [16], [24].

The Bland-Altman analysis is a measure of agreement between two instruments measuring on a continuous scale [17]. In the current analysis, the difference was evenly distributed across the range of average values, thereby supporting no bias in differences between high versus low quantification values. Regression analysis revealed no significant correlation between differences in log-transformed values with average values in the Bland-Altman plot.

HIV-1 genetic diversity is a key challenge to the development of HIV-1 quantitation assays [13], [25]–[27]. Subtype AE and B, the two predominant strains in Singapore, constitute about 45% each in a study of recent seroconverters [28]–[30]. Two previous studies from Thailand (a region where subtypes AE and B predominate), have described clinically validated in-house assays in 105 and 50 HIV-1 positive samples [31], [32]. However, both assays lacked internal controls, an important feature as inhibition rates can be as high as 3.7% in large-scale validations [16]. In contrast, inhibition was noticed in only 1 patient sample in the current evaluation. While it is acknowledged that extension of the Sing-IH assay to other subtypes would require clinical validation across a larger diversity of clinical samples [13], the ability to detect all subtypes in the WHO Reference Panel except group O suggests that SingIH is a suitable low-cost option for resource-limited settings.

The Sing-IH assay has several theoretical limitations to address in future development. Firstly, the sensitivity threshold of Sing-IH at 126 copies/mL is considerably higher than other commercial kits, whose detection threshold ranges from 20 to 50 copies/mL [33], [34]. As such, viral load values that fall within 50 to 200 copies/mL would be reported as being less than 200 copies/mL, but detectable, as recommended by the DHHS guidelines [35].

The actual clinical implications of viral loads that fall in the range of 50 copies to 200 copies/mL are currently unclear. Although some studies suggests that low level viremia may indicate virologic failure [36], there are studies that showed otherwise [37]. Clinically, transient elevations of viral load below 200 copies/mL have been defined as “blips” which are not associated with treatment failure or resistance emergence [38], [39]. There is no evidence that HIV-1 replicates and evolves when RNA levels are below this threshold and the observation that virologic failure was uncommon once viremia reached these levels [37]. Repeating values in 3 to 6 months at these levels to monitor for persistent viremia would be recommended.

Secondly, the extraction process is uncontrolled as in many in-house assays, where internal control is spiked into the PCR master mix instead of patient plasma in the beginning. To control for the extraction and reverse transcription, Sing-IH utilized HIV-1 Acrometrix standards and a commercially available positive control, which were subjected to nucleic acid extraction and amplification along with the patient specimens in each run. In the event where any of the standard or control results exceed the established validity range of the sample, all of the specimens and controls from the run must be retested, beginning with the sample preparation.

In conclusion, we developed a reliable, easy to use assay to increase access for reliable monitoring of response to therapy in regions where subtype B and AE predominate. The total cost of assay reagents is relatively low, at less than US$10 per reaction (excluding extraction, equipments, manpower and miscellaneous costs, Table S1) making it highly affordable to regions with resource-limited settings, which face cost hurdles in efforts to scale up viral load monitoring. This assay is currently implemented into routine testing in Singapore, and has routinely participated in an international proficiency-testing programme, TAQAS.

## Supporting Information

Table S1 **Breakdown of Actual Cost Required for Sing-IH assay's Operation.**
(DOC)
