# Supplementary material for: Clinical Evaluation of a Low Cost, In-House Developed Real-Time RT-PCR Human Immunodeficiency Virus Type 1 (HIV-1) Quantitation Assay for HIV-1 Infected Patients
Source: PLoS One. 2014 Mar 6;9(3):e89826. doi: 10.1371/journal.pone.0089826 (PMC3945479; doi:10.1371/journal.pone.0089826)
Supplement: Table S1 — Breakdown of Actual Cost Required for Sing-IH assay's Operation. (DOC) [file pone.0089826.s001.doc]

Supplementary Table 1: Breakdown of Actual Cost Required for Sing-IH assay’s Operation.

| **Estimated cost per sample** | **USD $** |
| --- | --- |
| Sample collection | 5.23 |
| RNA extraction | 11.46 |
| Amplification | 9.30 |
| Miscellaneous cost (eg electricity and administrative work) | 2.00 |
| **Total** | **27.99** |
